# Efficient In Planta Induction of Transgenic Hairy Roots in *Macadamia* Seedlings and Mature Trees Using Visual Reporters

**DOI:** 10.3390/plants15162418

**Published:** 2026-08-07

**Authors:** Yi Mo, Yu-Chong Fei, Xi Tian, Yujie Luo, Kai Lin, Meng Li, Jiajing Xu, Yuqi Pang, Yongwei Wu, Kuipeng Li, Liming Zeng, Sijie Huang, Zeng-Fu Xu

**Affiliations:** 1 Guangxi Key Laboratory of Forest Ecology and Conservation, Guangxi Colleges and Universities Key Laboratory for Cultivation and Utilization of Subtropical Forest Plantation, School of Forestry, Guangxi University, Nanning 530001, China; 2109401003@st.gxu.edu.cn (Y.M.); 2409392052@st.gxu.edu.cn (X.T.);; 2 Guangxi Key Laboratory of Quality and Safety Control for Subtropical Fruits, Guangxi Subtropical Crops Research Institution, Nanning 530001, China

**Keywords:** *Macadamia*, hairy roots, visual reporters, in planta, air layering

## Abstract

Macadamia (*Macadamia* spp.) is an economically important nut crop whose severe recalcitrance to genetic transformation substantially hinders progress in functional genomics and molecular breeding. To overcome this critical technical bottleneck, this study established a highly efficient and broadly applicable in planta hairy root genetic transformation system with integrated visual screening. This system utilizes an *Agrobacterium rhizogenes*-mediated transformation method, employing multiple visual reporter gene systems (*DsRed2*, *eGFP*, *RUBY*, and *AtPAP2*) to achieve antibiotic-independent and non-destructive screening of transgenic roots. Notably, the system innovatively incorporates the air layering (marcotting) technique to extend in planta genetic transformation to branches of mature trees in the field. By circumventing the stringent sterile conditions required for conventional in vitro tissue culture, this approach achieves a largely genotype-independent transformation across open-pollinated seedlings with diverse genetic backgrounds (A4, GR1, HAES900, and O.C.). The transgenic hairy root induction frequencies ranged from 39.25% to 47.38%, although the GR1 genotype exhibited a notable developmental stage-dependent decline in transformation efficiency. Furthermore, transgenic hairy roots were successfully induced on mature tree branches, with a maximum induction rate of 28.2%. Gene expression analyses confirmed the stable, high-level expression of the target transgenes in all the transgenic hairy root lines. This in planta transformation system provides a reliable in vivo experimental platform for the rapid functional validation of candidate genes and the investigation of root biology in *Macadamia*. Moreover, it establishes a novel strategy for plant regeneration via root-to-shoot organogenesis, offering a promising avenue for the genetic improvement of recalcitrant woody plants.

## 1. Introduction

Macadamia (*Macadamia* spp.), a woody nut tree species of high global economic value, is renowned for its premium, health-promoting kernels and has emerged as one of the most lucrative crops in the global tree nut market [1]. Cultivated primarily in major exporting countries such as Australia, South Africa, Kenya, and China, the global production of macadamia has expanded rapidly, securing a critical and highly competitive position alongside other major nut crops like almonds and walnuts [2,3]. In 2020, the global cultivation area for macadamia reached 6.05 million acres, of which China accounted for the largest share at 4.7 million acres, making it the world’s leading producer [3]. Despite its economic prominence, macadamia cultivation faces severe yield penalties and compromised kernel quality driven by complex agronomic challenges. Specifically, production is substantially threatened by devastating fungal pathogens and destructive insect pests, as well as increasing abiotic stresses including drought and extreme temperature fluctuations [2].

To address these critical yield-limiting factors, rapid advancements in molecular biology research and genomics-assisted breeding are driving the genetic improvement of this species [4,5,6]. However, constrained by the inherent biological characteristics of perennial woody plants, coupled with the prolonged, inefficient, and highly contamination-prone in vitro tissue culture processes, *Macadamia* has long lacked a highly efficient genetic transformation system. This deficiency severely impedes the functional validation of key genes and molecular improvement in this species [7]. Consequently, developing a rapid and efficient alternative transformation system that circumvents cumbersome aseptic tissue culture procedures remains an urgent priority.

In recent years, *Agrobacterium rhizogenes*-mediated hairy root composite plant systems have emerged as a highly efficient strategy for gene function studies in woody plants [7,8]. Unlike conventional tissue culture, the in planta *A. rhizogenes* inoculation method under non-sterile conditions enables researchers to perform transformations directly on intact plants grown in greenhouses or natural habitats. This strategy not only effectively circumvents the stringent sterility requirements and the risk of somaclonal variation inherent in tissue culture processes but also facilitates the generation of transgenic roots harboring target genes within a matter of weeks. Consequently, this significantly shortens the timeframe required for functional validation compared with traditional stable genetic transformation, providing a robust experimental platform for investigating root development, nutrient acquisition, and plant-microbe interactions [8,9].

Despite the substantial advantages of the in planta hairy root system, its widespread application remains constrained by several technical factors. First, the rapid, accurate, and non-destructive identification of positive transgenic hairy roots—without reliance on antibiotic or herbicide selectable markers—poses a formidable technical challenge. Traditional reporter systems, such as GUS staining, typically necessitate tissue fixation and chromogenic substrate incubation, thereby precluding continuous in vivo monitoring of the identical transformed roots [10]. The introduction of visual reporter genes—including fluorescent proteins [11,12] and systems capable of producing macroscopically visible pigments (e.g., the betalain-based reporter *RUBY* and the anthocyanin biosynthetic regulator *AtPAP2*) [13,14,15]—enables the non-destructive identification and dynamic tracking of transformation events. Second, the susceptibility of tissues to *Agrobacterium* infection is highly genotype-dependent, and varies significantly across different species and developmental stages [16]. This host-dependency frequently results in substantial fluctuations in transformation efficiency and even constitutes a critical limiting factor for the establishment of highly efficient hairy root systems in certain woody plants.

In light of these considerations, the present study established and systematically evaluated an *Agrobacterium rhizogenes*-mediated in planta hairy root induction system for *Macadamia*. To assess the impact of host genotype on transformation efficiency, we systematically compared the differential responses to hairy root induction across distinct *Macadamia* genotypes and validated the applicability of the system in both seedlings and 5-year-old branches. Furthermore, the scope of transformation recipients was extended to mature trees, successfully inducing the generation of transgenic hairy roots on the branches of adult trees via the air layering (marcotting) technique [17,18,19]. To facilitate the rapid identification of positive transgenic roots, we evaluated the efficacy of several visual reporters—namely *DsRed2*, *eGFP*, *RUBY*, and *AtPAP2*—in the induced hairy roots and subsequently analyzed the expression profiles of the transgenes within these positive events. Ultimately, the establishment of this system provides a robust tool for accelerating functional studies of root-related genes in *Macadamia*, while simultaneously serving as a valuable reference for developing hairy root transformation protocols in other recalcitrant woody plant species.

## 2. Results

### 2.1. Establishment of an Agrobacterium rhizogenes-Mediated Hairy Root Genetic Transformation System in Macadamia Hypocotyls

**Figure 1:**
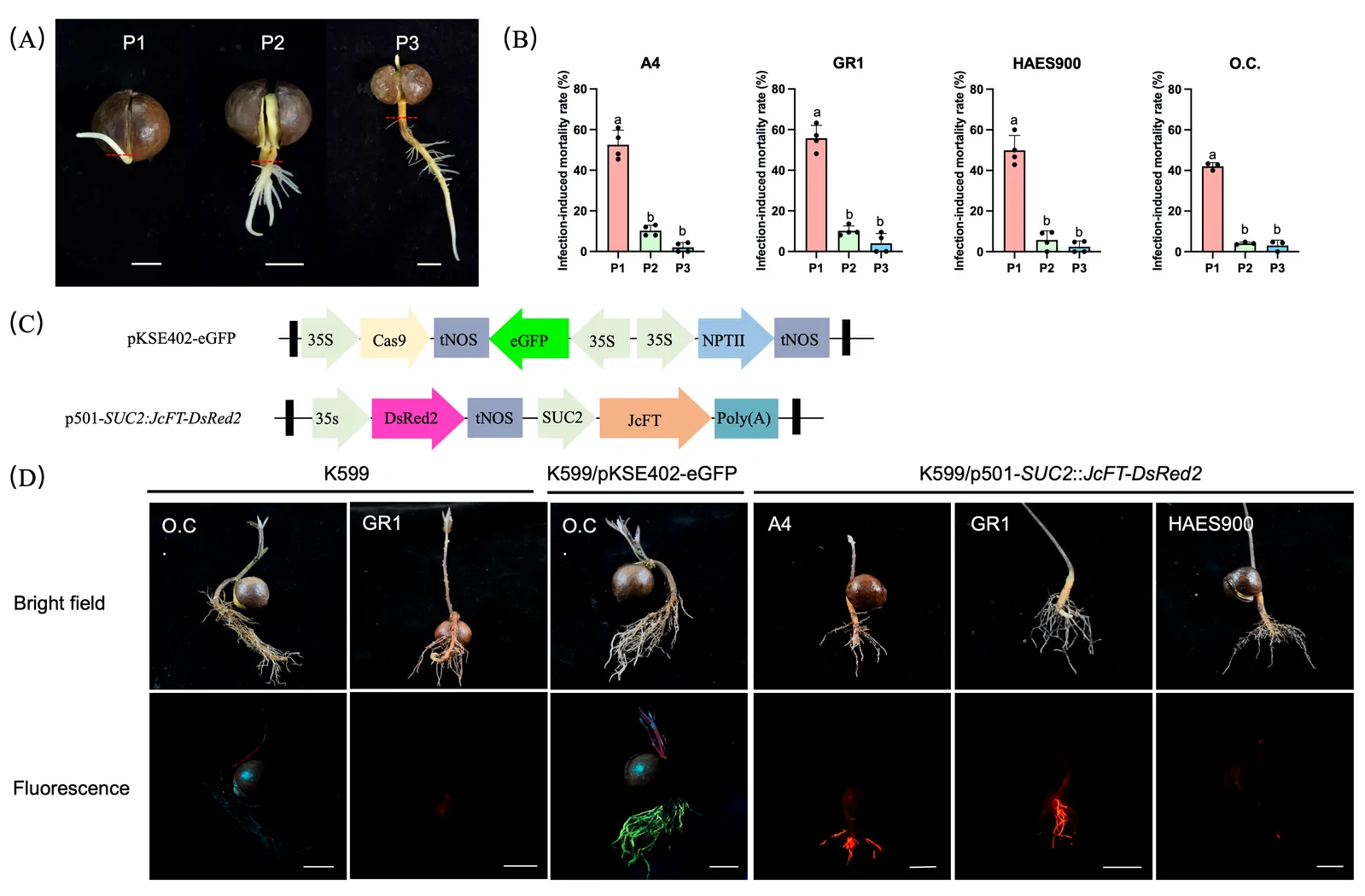
High-efficiency induction and identification of positive transgenic hairy roots in *Macadamia* seedlings. (**A**) Three distinct developmental stages (P1, P2, and P3) of *Macadamia* seedlings utilized for transgenic hairy root induction. The red dashed line indicates the excision site on the hypocotyl, which concurrently serves as the site for *Agrobacterium* inoculation. Scale bar = 2 cm. (**B**) Infection-induced mortality rates of four *Macadamia* genotypes (A4, GR1, HAES900, and O.C.) following treatments at different developmental stages (P1–P3). Infection-induced mortality rate = (number of dead plants post-inoculation/total number of inoculated plants) × 100%. The error bars represent the standard deviation (SD). Different lowercase letters above the bars indicate statistically significant differences among treatments at *p* < 0.05 (one-way ANOVA with Tukey’s HSD post hoc test on arcsine-square-root transformed proportion data). Untransformed percentages are shown in the figure. Raw data are provided in Supplementary Table S1. (**C**) Schematic representations of the T-DNA regions of the expression vectors utilized in this study: pKSE402-eGFP (harboring a 35S promoter-driven eGFP reporter gene) and p501-*SUC2*::*JcFT*-DsRed2 (harboring a 35S promoter-driven DsRed2 reporter gene). (**D**) Phenotypic observations of the induced positive transgenic hairy roots across different seedling genotypes inoculated with *A. rhizogenes* strain K599 harboring distinct expression vectors. Imaging was performed under bright field (upper panels) and fluorescence (lower panels). The K599 strain lacking the fluorescent reporter constructs served as the wild-type negative control. The fluorescence signals of the eGFP and DsRed2 proteins were detected under blue excitation light (approximately 440–460 nm) for O.C. and green excitation light (approximately 520–560 nm) for GR1, respectively. Scale bar = 2 cm.

Owing to their robust potential for cell division and differentiation, hypocotyls are frequently utilized as ideal recipient tissues for hairy root induction. However, the developmental stage of the recipient tissue directly determines both its genetic transformation efficiency and its tolerance to *Agrobacterium* infection. To this end, we evaluated *Macadamia* hypocotyls at three distinct developmental stages: P1 (early germination, characterized by the emergence of the radicle and hypocotyl through the seed coat), P2 (elongation of the hypocotyl and radicle, accompanied by lateral root emergence), and P3 (significant elongation of the hypocotyl and radicle, with pronounced lignification observed in the hypocotyl) (Figure 1A). These three stages collectively represent the developmental progression of the hypocotyl from a tender, juvenile state to maturation and aging.

Following radicle excision and subsequent inoculation with *Agrobacterium rhizogenes*, hypocotyls at distinct developmental stages exhibited significantly different sensitivities to mechanical wounding and *Agrobacterium* infection. The infection-induced mortality rates of hypocotyls across the four cultivars (A4, GR1, HAES900, and O.C.) demonstrated a pronounced developmental stage-dependency (Figure 1B, Supplementary Table S1). At the highly juvenile P1 stage, *Agrobacterium* infection provoked severe tissue necrosis and shoot wilting, resulting in mortality rates as high as 42% to 56% among the cultivars. Upon progressing to the P2 stage, the tolerance of the recipient tissues to infection-induced stress markedly increased, leading to a drastic reduction in mortality rates to 4–10%, which represented a statistically significant difference compared with that in the P1 stage (*p* < 0.05). By the P3 stage, survival rates were maintained at remarkably high levels, with no significant difference from those observed at the P2 stage. These results indicate that juvenile recipients at the P1 stage are highly vulnerable to infection-induced stress, whereas seedlings at the P2 and P3 stages exhibit robust stress tolerance, rendering them highly suitable recipient materials for efficient hairy root induction.

To further validate the feasibility of the transformation system utilizing P2 and P3 stage recipients, seedling hypocotyls were independently inoculated with *Agrobacterium rhizogenes* strain K599 harboring the visual fluorescent reporter constructs pKSE402-eGFP and p501-*SUC2*::*JcFT*-DsRed2 (Figure 1C). Under specific excitation wavelengths, distinct eGFP green or DsRed2 red fluorescence signals were successfully detected in the transgenic hairy roots induced across various cultivars. In contrast, no fluorescence emission was observed in the negative control group inoculated with the empty K599 strain (lacking fluorescent reporter constructs) (Figure 1D). These imaging results provide visual confirmation of the efficient expression of transgenes within the induced roots, thereby demonstrating the reliability of the established hairy root induction system for *Macadamia*.

### 2.2. Optimization of the Hairy Root Genetic Transformation System in Macadamia Hypocotyls

**Figure 2:**
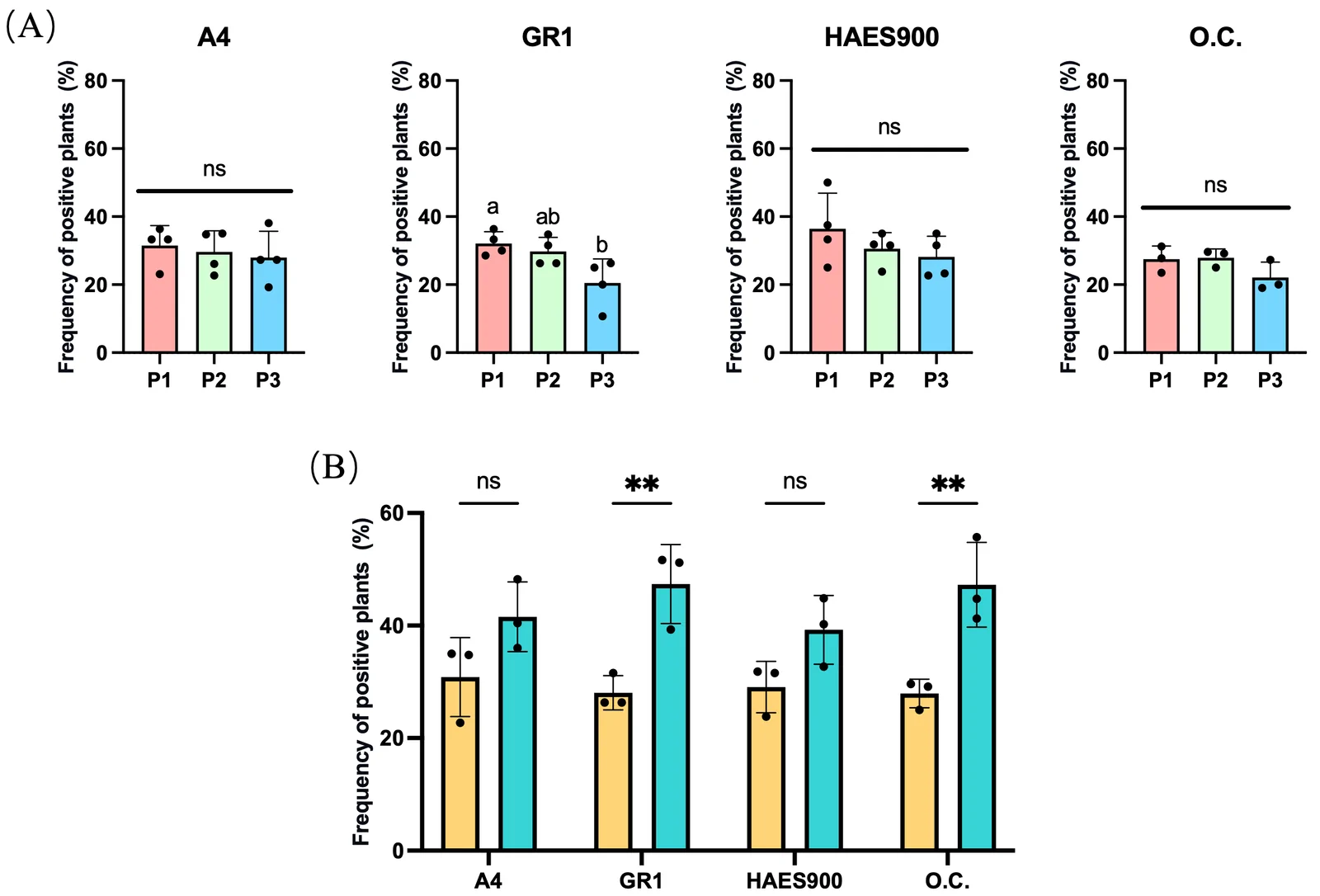
Effects of different developmental stages and inoculation treatments on the induction rates of positive transgenic *Macadamia* seedlings. (**A**) Frequencies (%) of positive transgenic plants induced across four *Macadamia* genotypes (A4, GR1, HAES900, and O.C.) at three distinct developmental stages (P1, P2, and P3). The error bars represent the standard deviation (SD), and the scatter points indicate independent biological replicates. Different lowercase letters above the bars indicate statistically significant differences among groups (*p* < 0.05), whereas ‘ns’ denotes no significant difference (*p* > 0.05; one-way ANOVA with Tukey’s HSD post hoc test on arcsine-square-root transformed proportion data). Untransformed percentages are shown in the figure. Raw data are provided in Supplementary Table S1. (**B**) Comparative effects of two distinct inoculation treatments (yellow bars represent the dip-inoculation method, DIP; blue bars represent the injection-inoculation method, INJ) on the frequencies (%) of positive transgenic plants across the four *Macadamia* genotypes. Asterisks (**) indicate highly significant differences between the two treatments (*p* < 0.01), whereas ‘ns’ denotes no significant difference (*p* > 0.05; two-way ANOVA with Sidak’s post hoc test for multiple comparisons on arcsine-square-root transformed proportion data). Untransformed percentages are shown in the figure. Raw data are provided in Supplementary Table S2.

To further evaluate the impact of the developmental stage on the genetic transformation efficiency, we quantified the positive hairy root induction rates across four cultivars (A4, GR1, HAES900, and O.C.) during the P1–P3 stages (Figure 2A, Supplementary Table S1). The results revealed that the transformation efficiencies of three cultivars—A4, HAES900, and O.C.—remained relatively stable from the P1 to P3 stages, ranging between 20% and 40%, with no statistically significant differences observed among the developmental stages (*p* > 0.05). In contrast, cultivar GR1 exhibited a pronounced developmental stage-dependency. Its positive induction rate at the P1 stage was significantly higher than that at the P3 stage (*p* < 0.05), indicating that the juvenile tissues of this genotype exhibit greater transformation potential, with transformation efficiency declining as seedling age increases.

In addition, this study compared the effects of two *Agrobacterium* inoculation methods—the dip-inoculation method (DIP) and the injection-inoculation method (INJ)—on hairy root induction efficiency. The results revealed (Figure 2B, Supplementary Table S2) that the positive transgenic root induction rates achieved via DIP ranged from 27.93% to 30.84%, whereas the rates obtained via INJ increased to 39.25–47.38%. Notably, in the GR1 and O.C. cultivars, the induction efficiency of the INJ method was significantly superior to that of the DIP method. This discrepancy may be attributed to the deeper mechanical wounding caused by needle injection, which penetrates the hypocotyl epidermis and forces the *A. rhizogenes* suspension directly into the internal vascular cambium and deeper parenchymal cells. Consequently, this physical delivery enhances both the number of accessible target cells and the probability of effective T-DNA integration. Taken together, the INJ method demonstrates superior broad applicability and high efficiency for the induction of positive transgenic hairy roots in *Macadamia*.

### 2.3. Application of Pigment-Synthesizing Reporter Genes in the Hairy Root Genetic Transformation of Macadamia

**Figure 3:**
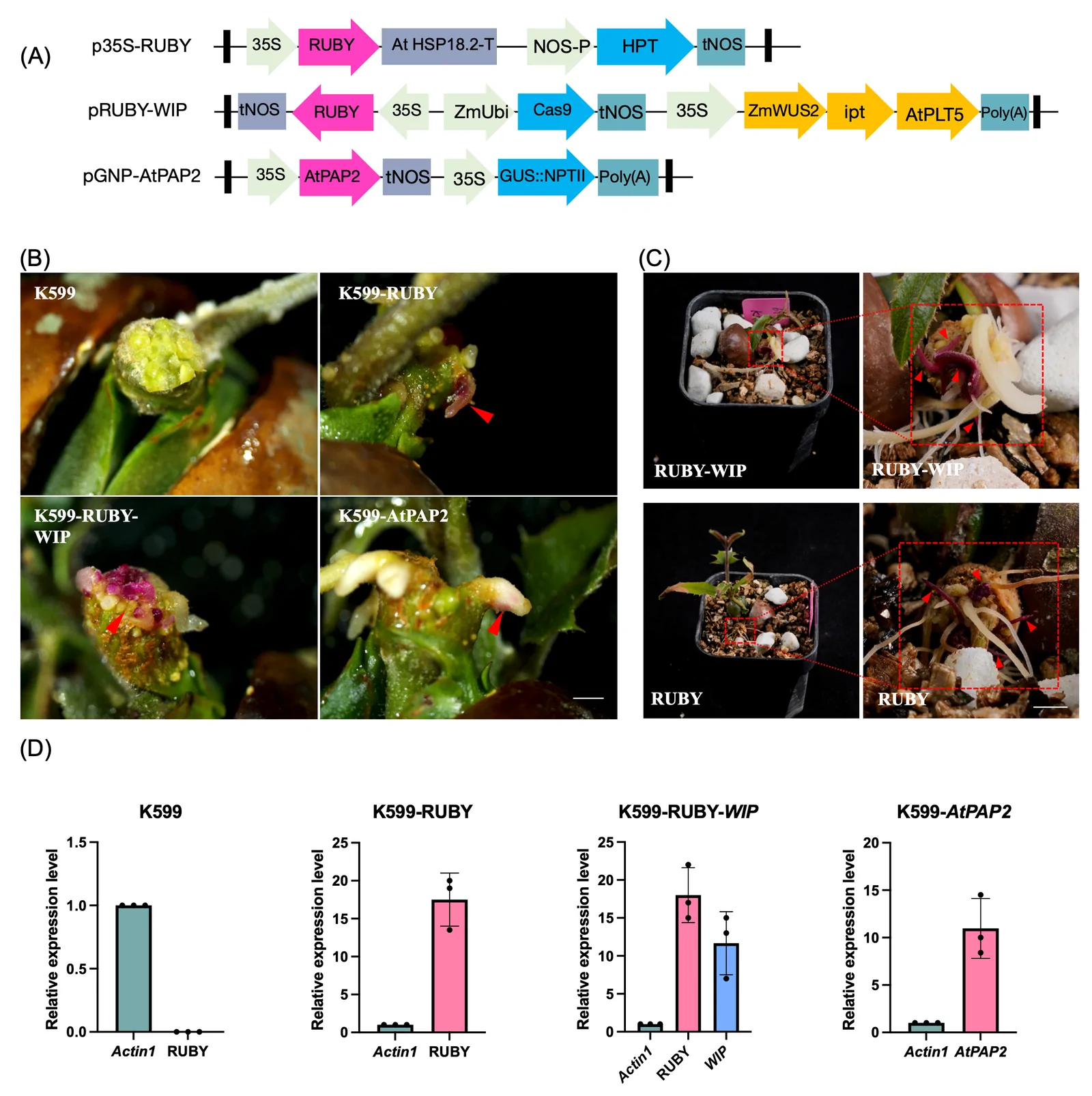
Identification and molecular validation of in planta hairy roots in *Macadamia* mediated by multiplex visual reporter systems. (**A**) Schematic representations of the T-DNA regions of the three expression vectors utilized in this study. (**B**) Early phenotypic observations of hairy roots induced at the wounding sites on *Macadamia* branches following inoculation with the wild-type *Agrobacterium rhizogenes* strain K599 and engineered strains harboring distinct expression vectors. Red arrows indicate distinct color changes. Scale bar = 2.5 mm. (**C**) Phenotypes of positive transgenic hairy roots developing on potted *Macadamia* seedlings. The red dashed box corresponds to the magnified view on the right, where red arrows explicitly indicate the positive transgenic roots exhibiting distinct red coloration within the soil matrix. (**D**) Quantitative analysis of target gene expression levels in hairy roots under different treatments via RT-qPCR. The relative expression levels of *RUBY*, the *WIP* developmental regulatory cassette, and the *AtPAP2* gene were assayed in hairy roots induced by the wild-type strain (K599) and strains harboring the p35S-RUBY, pRUBY-WIP, and pGNP-AtPAP2 vectors, respectively. *Actin1* was utilized as the internal reference gene. Error bars represent the standard deviation (SD) of independent biological replicates. The qPCR data in (**D**) are presented quantitatively to illustrate the relative expression trends.

To investigate the application efficacy of two visual pigment-synthesizing reporter systems—specifically those mediating betalain and anthocyanin biosynthesis—in regenerated roots derived from *Agrobacterium rhizogenes*-mediated in planta transformation, this study utilized the injection-inoculation method. Recipient tissues were independently inoculated with the empty K599 strain and K599 strains containing p35S-RUBY/pRUBY-WIP/pGNP-AtPAP2 (Figure 3A) to characterize the phenotypes of the resulting hairy roots and assess the expression profiles of the transgenes.

The results revealed that inoculation with the empty K599 strain induced a certain number of adventitious roots; however, these roots lacked any visual marker signals, representing typical adventitious roots elicited solely by the endogenous Ri plasmid (Figure 3B). Following inoculation with K599-RUBY, a subset of the induced roots exhibited a macroscopically visible dark red coloration. This phenotype is attributed to the production and accumulation of betalain pigments within the tissues driven by the expression of the *RUBY* reporter system, thereby demonstrating the successful expression of the exogenous betalain biosynthetic genes in the hairy root tissues (Figure 3B). In the case of the K599-RUBY-WIP treatment, both the induced roots and their associated calli similarly displayed distinct RUBY-mediated red signals (Figure 3B,C). Upon inoculation with K599-AtPAP2, a proportion of the induced roots exhibited a pale purple or light red phenotype (Figure 3B). This phenomenon occurs because the transcription factor *AtPAP2* is capable of activating the endogenous anthocyanin biosynthetic pathway, leading to the accumulation of anthocyanin-class pigments in the tissues. This result concurrently verifies the successful expression of the transgene in the induced roots. Subsequent gene expression analysis confirmed that these visually pigmented roots were positive transgenic roots (Figure 3D). Genomic PCR further confirmed the stable integration of the *RUBY* transgenes in a representative subset of the pigmented roots (Supplementary Figure S1). Collectively, these findings demonstrate that macroscopic, direct visual screening systems based on the pigment-synthesizing reporters *RUBY* and *AtPAP2* enable the early, non-destructive, and intuitive identification of transgenic roots in the absence of antibiotic selection pressure.

### 2.4. Establishment of an A. rhizogenes-Mediated Hairy Root Genetic Transformation System in Mature Macadamia Branches via the Air Layering Technique

**Figure 4:**
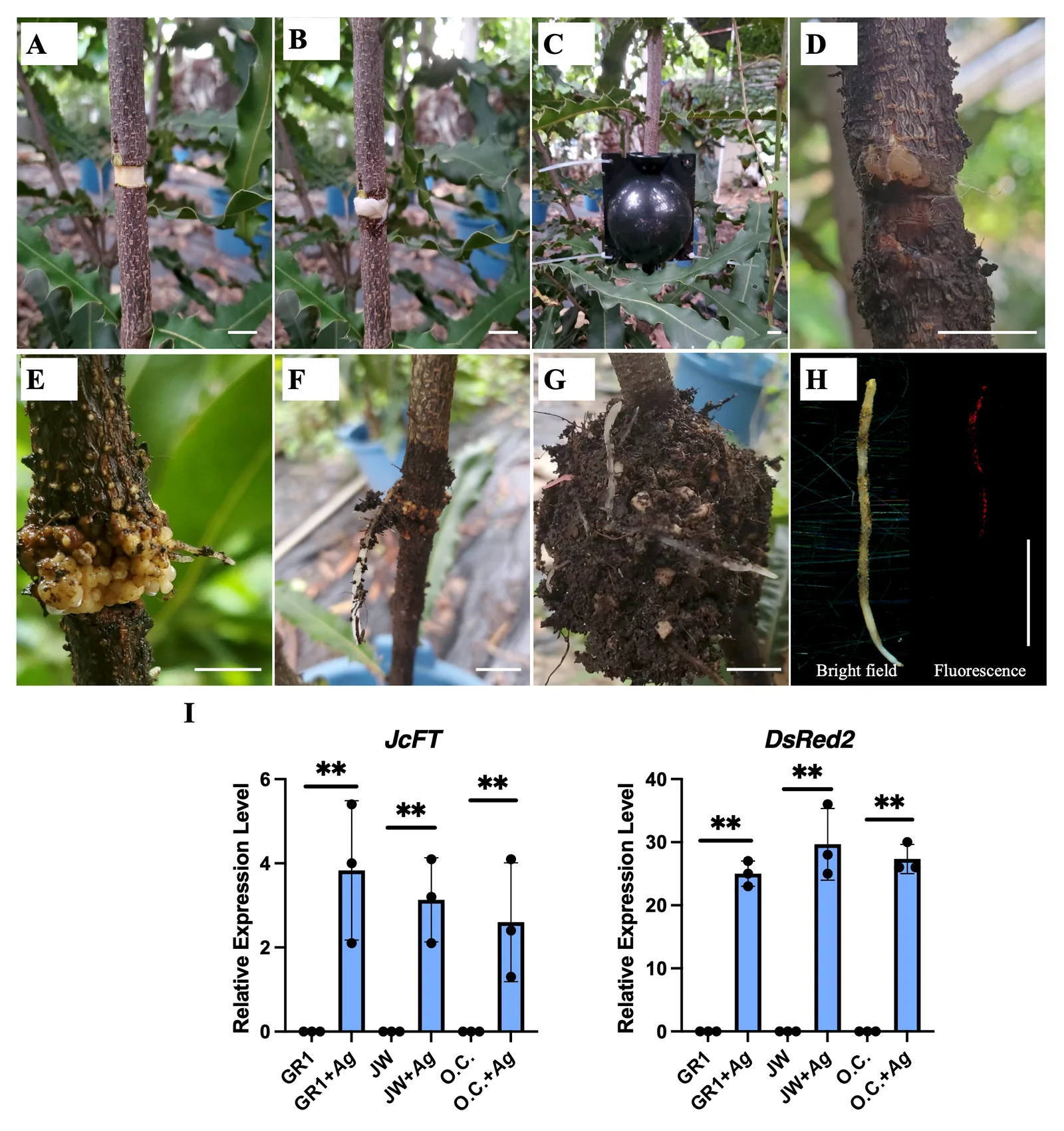
In planta induction and molecular validation of hairy roots on mature *Macadamia* branches via air layering. (**A**–**C**) Operational workflow of in planta hairy root transformation integrated with the air layering technique. (**A**) Physical girdling of a mature branch. (**B**) Application of *Agrobacterium rhizogenes* bacterial paste harboring an expression vector (e.g., p501-*SUC2*::*JcFT*-DsRed2) to the girdled wounding site. (**C**) Wrapping and light-exclusion treatment utilizing a rooting ball filled with a moisture-retaining substrate. (**D**–**G**) Dynamic morphological evolution of tissues at the wounding site during the induction process. (**D**) Early formation of cortical callus. (**E**) Massive proliferation of the callus accompanied by the emergence of hairy root primordia. (**F**) Elongation growth of the induced positive hairy roots. (**G**) Observation of the extensive hairy root system developed within the substrate upon removal of the rooting ball. Scale bars in (**A**–**G**) = 1 cm. (**H**) Phenotypic observations of the induced hairy roots under bright-field (left) and fluorescence (right) conditions. Red fluorescence indicates the successful expression of the *DsRed2* reporter gene within the root vascular tissues. Scale bar = 1 cm. (**I**) Quantitative analysis of the relative expression levels of the target gene *JcFT* and the reporter gene *DsRed2* across three mature *Macadamia* genotypes (GR1, JW, and O.C.) via RT-qPCR. Wild-type branches without *Agrobacterium* inoculation served as the control group, whereas ‘+Ag’ denotes the experimental group inoculated with *A. rhizogenes*. Error bars represent the standard deviation (SD) of independent biological replicates. Asterisks indicate highly significant differences between the control and transformed groups (*p* < 0.01; one-way ANOVA with Tukey’s HSD post hoc test).

The air layering technique provides a highly efficient method for vegetative propagation by promoting root formation on mature branches. It not only facilitates the rapid clonal multiplication of elite cultivars [17,18,19], but also holds significant potential for applications in genetic transformation and molecular breeding. To investigate the stimulatory effects of *Agrobacterium rhizogenes* on root induction in mature *Macadamia* branches, robust and disease-free 5-year-old grafted plants (cultivars: GR1, JW, and O.C.) were selected as experimental materials. The target branches were subjected to girdling, with the phloem completely removed to create an artificial wounding interface. The wound sites were subsequently wrapped in absorbent cotton saturated with a bacterial suspension of *A. rhizogenes* strain K599/p501-*SUC2*::*JcFT*-DsRed2 (Figure 1C), which was then firmly secured using an air layering rooting box (Figure 4A–C). At 17 days post-treatment, the tissue immediately above the girdling site began to swell, accompanied by macroscopically visible initial callus formation (Figure 4D). Over time, the calli underwent further proliferation and subsequent differentiation into root primordia. By 30 days post-treatment, a substantial mass of compact callus had developed at the wounding site, which was concurrent with the distinct emergence of adventitious roots (Figure 4E). At 40 days post-treatment, the initially emerged roots entered a rapid elongation phase, during which time the length of the roots significantly increased (Figure 4F). By 65 days post-treatment, the number of roots had further increased, culminating in the formation of a relatively well-developed root system architecture (Figure 4G). Subsequent sampling and observation under a fluorescence stereomicroscope revealed distinct red fluorescence signals within the induced roots (Figure 4H).

To validate the transcriptional activity of the transgenes within the mature-stage transformation system, the induced transgenic roots from the three cultivars were analyzed utilizing RT-qPCR (Figure 4I). The results demonstrated that following inoculation with *Agrobacterium rhizogenes* (Ag), the relative expression levels in the GR1 + Ag, JW + Ag, and O.C. + Ag treatments were markedly higher than those in their respective control groups. Specifically, compared with those of the corresponding uninoculated controls, the transcript abundances of the transgenes *JcFT* and *DsRed2* were significantly elevated (*p* < 0.01). Notably, although the transcription of the floral-promoting gene *JcFT* was high in the transgenic roots, the morphological apical end of the rooted branches failed to exhibit any floral bud differentiation phenotype during the observation period (within 2 months post-inoculation).

### 2.5. Optimization of Conditions for the Air Layering-Based Hairy Root Genetic Transformation System in Macadamia

**Figure 5:**
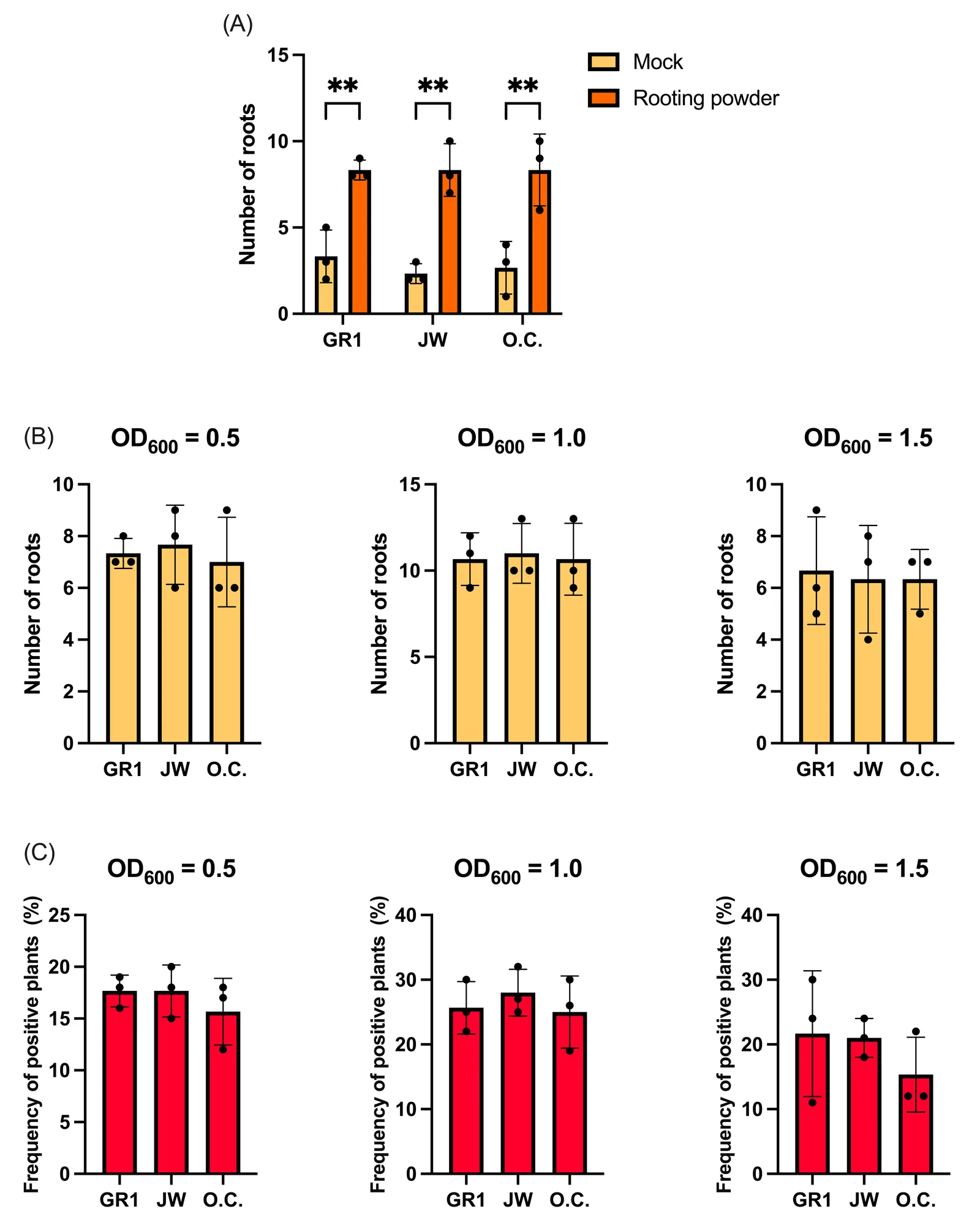
Optimization of *Agrobacterium rhizogenes* inoculation density for air layering-based rooting and transformation efficiency in mature *Macadamia*. (**A**) Effects of control treatments on adventitious root induction across three mature *Macadamia* cultivars (GR1, JW, and O.C.), where ‘Mock’ served as the blank control and ‘Rooting powder’ denotes the exogenous rooting hormone treatment. (**B**) Effects of varying *A. rhizogenes* suspension concentrations (OD_600_ = 0.5, 1.0, and 1.5) on adventitious root induction. The columns represent the average number of roots generated at the air layering sites. (**C**) Effects of varying *A. rhizogenes* suspension concentrations (OD_600_ = 0.5, 1.0, and 1.5) on adventitious positive transformation efficiency. Black scatter points signify independent biological replicates, and error bars represent the standard deviation (SD). In (**A**,**C**), asterisks indicate significant differences between the two treatments (** *p* < 0.01; one-way ANOVA with Tukey’s HSD post hoc test on arcsine-square-root transformed proportion data). Untransformed percentages are shown in the figure. Raw data are provided in Supplementary Table S3.

To systematically optimize the transformation efficiency of the air layering-based system, this study evaluated the effects of various treatment conditions on the number of roots generated per individual branch and the transformation frequencies across three cultivars (GR1, JW, and O.C.). Under mock-inoculated control conditions (without *Agrobacterium*), all three cultivars (GR1, JW, and O.C.) were capable of producing adventitious roots, yielding averages of 3.3, 2.3, and 2.7 roots per branch, respectively (Figure 5A). However, the frequency of positive transgenic plants was essentially zero, indicating that while the air layering technique inherently facilitates root induction under natural conditions, no genetic transformation events occurred (Figure 5A, Supplementary Table S3). Following treatment with exogenous rooting powder, the number of adventitious roots uniformly increased to an average of 8 per branch across all three cultivars (Figure 5A, Supplementary Table S3). These findings demonstrate that although plant growth regulators can effectively stimulate adventitious root formation, they similarly do not yield any positive transgenic roots.

In the *Agrobacterium* inoculation treatments, varying the bacterial suspension concentration resulted in pronounced differences in both the number of induced roots and the frequency of positive transgenic plants. At a bacterial cell density of OD_600_ = 0.5, all three cultivars (GR1, JW, and O.C.) generated a certain proportion of positive transgenic roots (17.7%, 17.7%, and 15.7%, respectively) (Figure 5B, Supplementary Table S3). This finding indicates that although a relatively low concentration of *Agrobacterium* can achieve effective infection, the overall transformation efficiency remains relatively low. As the bacterial concentration increased to OD_600_ = 1.0, the positive transgenic frequencies across the three cultivars significantly elevated (to 26.0%, 28.2%, and 24.8%, respectively) (Figure 5B, Supplementary Table S3), while the number of induced roots concurrently remained robust. This suggests that at this specific cell density, an optimal balance between *Agrobacterium* infection efficiency and plant tissue viability is achieved, thereby yielding a superior transformation efficiency. Upon further elevating the bacterial density to OD_600_ = 1.5, the positive transgenic frequencies did not exhibit any further substantial enhancement compared with those at OD_600_ = 1.0; instead, a slight decrease was observed in certain cultivars (dropping to 21.6%, 21.0%, and 15.3%, respectively) (Figure 5B, Supplementary Table S3). It is postulated that excessive bacterial concentrations may inflict severe biological stress on plant tissues, consequently impairing overall root induction and transformation outcomes. Furthermore, no significant genotype-dependent differences were observed among the distinct recipient cultivars across these treatments.

Taken together, these findings indicate that the cell density of the Agrobacterium suspension profoundly affects the transformation efficiency of the air layering-based hairy root induction system. Under the present experimental conditions, an OD_600_ of 1.0 represented the relatively optimal inoculation concentration. It is capable of securing a robust number of induced roots while concurrently yielding a high proportion of positive transgenic hairy roots, thereby establishing an optimized parameter framework for downstream applications.

## 3. Discussion

### 3.1. Overcoming the Age Limitation of Transformation in Woody Plants by Integrating Air Layering with Agrobacterium rhizogenes

In the genetic transformation of woody fruit trees, the strict dependence on juvenile explants has long constituted an insurmountable bottleneck [16,20]. Due to a pronounced decline in cellular totipotency, mature tree tissues are exceedingly recalcitrant to de novo organogenesis via conventional induction protocols [16,20]. Recently, a high-pressure propagation breeding (HPPB)-mediated transformation system was developed for citrus rootstocks, successfully integrating *Agrobacterium tumefaciens* infection with an air-layering strategy [19]. While this elegantly demonstrated the feasibility of combining air-layering with in planta transformation in woody plants, our system advances this concept in three critical dimensions. First, unlike citrus, macadamia (Proteaceae) is notoriously recalcitrant to transformation; prior to this study, no efficient hairy root induction system existed for this species. Second, moving beyond the reliance on visual markers alone (such as *GFP* and *RUBY*), our construct incorporates an *FT* overexpression cassette alongside the *DsRED2* reporter. This integration enables the simultaneous, direct evaluation of flowering-related gene function within the transgenic hairy roots. Finally, and most significantly, whereas the citrus HPPB system was validated on 2- to 3-year-old juvenile seedlings, our protocol was established exclusively on grafted, reproductively mature, field-grown trees (Figure 4 and Figure 5). By inducing transgenic hairy roots directly on adult branches, our approach offers a rapid and highly relevant platform for in planta functional genomics that closely aligns with the practical realities of tree crop breeding.

The endogenous hormonal homeostasis inherent in mature woody tissues typically precludes cellular dedifferentiation [7]. Conversely, the physical intervention of air layering (girdling) artificially generates a localized microenvironment enriched with auxins and carbohydrates [21,22]. Building upon this foundation, in planta inoculation with *A. rhizogenes* introduces *rol* genes that amplify hormonal signaling, ultimately resulting in cell fate reprogramming within these exceptionally recalcitrant mature tissues [8,23]. This molecular-level cell fate reprogramming successfully breaches the transformation recalcitrance barrier in mature *Macadamia* tissues, rendering direct in vivo gene functional validation in adult trees feasible. Importantly, our mature-tree evaluations relied on multiple branches sourced from a limited number of individual trees (Table S3). Because these branch replicates share a common physiological background, potential confounding effects arising from tree-to-tree variation cannot be completely excluded.

Germplasm innovation in woody plants predominantly relies on conventional seedling breeding, which is severely constrained by a prolonged juvenile phase prior to initial flowering, thus significantly impeding genetic improvement [24,25]. Classical floral induction models indicate that the FLOWERING LOCUS T (FT) protein undergoes long-distance transport from the leaves to the shoot apical meristem (SAM) via the phloem. It subsequently interacts with the bZIP transcription factor *FD*, which is specifically expressed within the SAM, to orchestrate the floral transition program [26,27]. In the woody plant *Jatropha curcas*, the JcFT protein can be robustly translocated from a transgenic rootstock to a non-transgenic scion, thereby promoting early flowering in the scion [28,29]. Furthermore, FT synthesized in transgenic hairy roots has also been shown to be translocated from the subterranean root system to aerial shoots [30]. In the present study, despite RT-qPCR confirming the high-level transcription of *JcFT* within the air layering-induced positive hairy roots (Figure 4I), the SAM of the corresponding layered branches failed to exhibit the anticipated floral bud differentiation. This observation aligns with similar findings in the gymnosperm woody plant *Pinus tabuliformis* [30]. The responsiveness of the SAM to florigenic signals in woody plants is generally subject to stringent seasonal and epigenetic regulation [31,32]. Consequently, we hypothesize that the lack of early flowering stems from either an insufficient concentration of FT protein at the target site or a lack of developmental competence in the recipient tissues. Specifically, long-distance FT transport mechanisms may differ across heterologous systems, and the immense biomass of mature macadamia branches could dilute the translated JcFT protein prior to SAM arrival. Additionally, responding to florigenic signals might require mature tissues to undergo profound epigenetic remodeling, such as repressing FLOWERING LOCUS C (FLC) homologs. As these explanations are not directly supported by the data in the present study, they represent speculative but highly probable mechanisms that warrant further investigation.

### 3.2. Integration of the in Planta Non-Sterile System with Visual Transgenic Screening Systems

Traditional tissue culture processes are frequently accompanied by severe tissue browning, exceptionally high contamination rates, and the potential risk of somaclonal variation [7]. The in planta transformation system established in this study completely circumvents these cumbersome aseptic procedures, substantially shortening the experimental duration to an average of only 30 days from inoculation to the emergence of positive roots. To comprehensively evaluate the broad applicability and stability of this transformation protocol, we simultaneously incorporated four distinct types of reporter genes (*DsRed2*, *eGFP*, *RUBY*, and *AtPAP2*) into the *Macadamia* hairy root induction system (Figure 1C and Figure 3A). The results demonstrate that this hairy root induction method functions independently of specific vector backbones and therefore has broad application potential.

In contrast to the conventional GUS staining assay, which relies on destructive biochemical reactions [10], the visual systems employed in this study facilitate continuous screening within intact living plants. The fluorescent systems (eGFP and DsRed2) satisfy the precise requirements for subcellular localization and high-resolution histological observation of hairy roots [11,12]. They are capable of effectively bypassing interference from endogenous tissue pigments under specific excitation wavelengths, thus conferring high-resolution advantages in tissue anatomy and fine-scale localization studies. However, their reliance on fluorescence microscopy equipment limits their convenience for field environments and high-throughput macroscopic screening; furthermore, fluorescent signals are prone to attenuation in aging root systems.

By comparison, pigment-synthesizing reporter genes enable non-destructive, macroscopic visual screening. Notably, within the root system investigated in this study, the *RUBY* reporter yielded a more distinct visual signal than *AtPAP2*. The underlying mechanism is that the *RUBY* system, by introducing a complete suite of betalain biosynthetic enzymes, directly utilizes endogenous tyrosine to synthesize the pigment, rendering it independent of the secondary metabolic background of the recipient plant. Conversely, as a transcription factor, *AtPAP2*-mediated anthocyanin accumulation is highly dependent on the coordinated expression of endogenous anthocyanin biosynthetic pathway-related genes within the recipient. The root-specific gene expression patterns in *Macadamia* likely restrict its coloration efficacy [14,15]. While pigment-based reporter systems like *RUBY* and *AtPAP2* offer the distinct advantage of convenient visual screening, their reliability can be compromised by stress-induced endogenous anthocyanin accumulation, which confounds signal interpretation in certain species. Furthermore, although *RUBY* provides excellent macroscopic visibility, we observed pigment degradation over prolonged cultivation periods. To mitigate these limitations, integrating pigment-based markers with fluorescence reporters or subsequent molecular validation is recommended to ensure the robust identification of transformants.

### 3.3. Overcoming Genotype Dependency to Construct a Broad-Spectrum Genetic Transformation Platform

Genotype dependency constitutes another major limitation restricting the widespread application of *Agrobacterium*-mediated transformation technologies [7]. Distinct *Macadamia* genotypes exhibit substantial variations in endogenous hormone levels, phenolic compound accumulation, recalcitrance to in vitro regeneration, and susceptibility to *Agrobacterium* infection, resulting in an exceedingly narrow applicability of conventional transformation methods [7,33]. The in planta transformation strategy employed in this study effectively exploits the robust endogenous homeostasis of intact plants as a buffer, thereby completely circumventing the extremely stringent conditions required for in vitro tissue culture [8,34]. Moreover, by proceeding via direct organogenesis, this system successfully bypasses the highly genotype-restricted regeneration bottleneck [7,16].

The seedlings utilized in this study were derived from the germination of open-pollinated seeds and thus possessed highly diverse genetic backgrounds. The results demonstrated that although distinct genotypes exhibited slight variations in the timing of root initiation because of differences in their initial endogenous hormone levels (Figure 1A), the *rol* gene cluster harbored by *A. rhizogenes* profoundly amplified the sensitivity of the recipient cells to hormones, thereby minimizing the differential responses among genotypes [8]. Consequently, upon the integration of the expression cassettes—with or without developmental regulatory factors—all the tested genotypes ultimately and successfully yielded positive transgenic hairy roots (Figure 1D; Figure 3B,C; Figure 4A–H; and Figure 5C). These findings demonstrate that our *A. rhizogenes*-mediated in planta hairy root induction strategy is robust and broadly applicable across genotypes. This system is no longer confined to a single model cultivar and may provide a broadly applicable platform for germplasm evaluation and gene function characterization across *Macadamia* and other recalcitrant woody plant species.

### 3.4. Limitations and Future Prospects of the Established Genetic Transformation System

It should be objectively recognized that the in planta transgenic hairy root induction system inherently produces composite (chimeric) plants, in which the root system is transgenic while the shoot system remains wild-type. Due to the untransformed nature of the reproductive primordia, this system is incapable of directly generating heritable transgenic progeny. Nevertheless, this intrinsic limitation does not preclude the system’s ability to generate stable transgenic materials. In fact, *Agrobacterium rhizogenes*-induced hairy roots exhibit vigorous proliferation and high cellular totipotency. These positive roots, rigorously identified via visual screening systems, serve as excellent explants for in vitro regeneration [35]. Moving forward, optimizing the in vitro hairy root regeneration system to induce de novo adventitious shoot differentiation from these positive roots will provide a highly promising alternative pathway. This approach could completely circumvent the recalcitrance associated with the regeneration of conventional *Macadamia* stem and leaf tissues, thereby facilitating the recovery of intact, stably transformed plants [35,36].

This composite plant system also provides a powerful platform for elucidating the molecular mechanisms underlying the unique biological traits and important agronomic characteristics of *Macadamia*. For example, by integrating multi-omics approaches such as spatial transcriptomics and metabolomics, this system can be used to dissect key biological processes, including the development of proteoid roots (cluster roots), the regulatory networks of efficient phosphorus acquisition, and root-microbe symbiotic interactions [37,38,39]. Furthermore, this system can serve as an efficient tool for functional validation of genes in macadamia roots, while also enabling rapid assessment of gene editing efficiency within the species [19]. It may function as a homologous system for evaluating the activity and off-target effects of gene editing tools such as CRISPR–Cas9. In this way, prior to committing months or even years to stable transformation, researchers can utilize transgenic hairy root systems to quickly validate sgRNA editing efficiency and potential off-target effects, thereby substantially reducing trial-and-error costs. Collectively, these applications will provide robust empirical support for future molecular breeding endeavors in macadamia.

## 4. Materials and Methods

### 4.1. Plant Materials

All five cultivars (A4, GR1, HAES900, JW and O.C.) are diploid (2n = 28). Plant materialsm were obtained from Guangxi Fusui Xiaguo Plantation Co., Ltd. (Fusui, China). The seedlings utilized in the experiments were derived from fully developed, mature seeds harvested from *Macadamia* germplasms A4, GR1, HAES900, and O.C. These specific germplasms were selected not only for their commercial significance but also for their representative and contrasting developmental phenotypes. Detailed information regarding the cultivars and seedlings is provided in Table 1 and Supplementary Tables S1 and S2. All seeds were obtained via natural open pollination. The seeds were submerged in clean water for approximately 3 to 4 days to ensure complete imbibition. Subsequently, the fully imbibed seeds were sown in trays filled with vermiculite and covered with a layer of the same substrate. The trays were then transferred to a plant growth chamber maintained at 26 °C with a 16-h light/8-h dark photoperiod. The vermiculite was kept adequately moist by daily spraying with water. Seed germination was continuously monitored, and subsequent experiments were initiated upon the evident elongation of the hypocotyls.

The air layering experiments were conducted under natural field conditions. Specifically, the trials were carried out from September to November 2024 in Nanning, Guangxi, China. During the experimental period, the ambient natural environment had an average temperature of approximately 24–25 °C and a relative humidity of 72–78% (https://zh.weatherspark.com), providing an optimal microclimate for adventitious rooting and hairy root induction. Healthy branches with stem diameters of approximately 1.0 to 1.5 cm were selected from 5-year-old grafted plants of three *Macadamia* cultivars (GR1, JW, and O.C.) cultivated in an orchard. Detailed information regarding the number of mature trees and the corresponding number of independent branches used for each cultivar is provided in Supplementary Table S3. A girdling knife was used to completely remove a circumferential strip of approximately 1 cm wide bark from each selected branch to facilitate the treatment.

### 4.2. Plasmid Vectors

The pKSE402-eGFP vector employed in this study harbors an enhanced green fluorescent protein (*eGFP*) reporter gene driven by the CaMV 35S promoter, along with the kanamycin resistance selectable marker gene *nptII* and the *Cas9* coding sequence [11]. It should be noted that although pKSE402 is typically utilized as a CRISPR/Cas9 binary vector, in this study, the empty vector was employed exclusively for its independent eGFP expression cassette to serve as a visual reporter for the transformation system. Since no specific gRNAs were designed or inserted into the construct, the inherent Cas9 protein lacked a genomic target, thereby ensuring that no gene editing events occurred during the process. The p501-*SUC2*::*JcFT*-DsRed2 vector contains a *JcFT* expression cassette driven by the phloem-specific *SUC2* promoter and an enhanced red fluorescent protein (*DsRed2*) reporter gene driven by the CaMV 35S promoter, while also carrying the *nptII* selectable marker [28,43,44]. The p35S-RUBY vector integrates the *RUBY* visual reporter system driven by the CaMV 35S promoter and carries the hygromycin resistance selectable marker gene *hpt* driven by the *nos* promoter [45]. The pRUBY-WIP vector comprises a 35S promoter-driven *RUBY* reporter module and a *ZmUbi* promoter-driven *Cas9* expression cassette, concurrently harboring a *WIP* developmental regulatory module driven by the 35S promoter. This *WIP* module consists of three tandemly arrayed genes: *ZmWUS2*, *ipt*, and *AtPLT5* [46]. The pGNP-AtPAP2 vector carries the anthocyanin accumulation regulatory factor *AtPAP2* driven by the CaMV 35S promoter and incorporates a *GUS::NPTII* fusion gene, thereby conferring dual functionalities for both histochemical assays and kanamycin resistance selection [14].

### 4.3. Genetic Transformation

A 2 mL aliquot of the *Agrobacterium rhizogenes* strain K599 liquid culture was centrifuged at 8000× *g* for 2 min to harvest the bacterial cells. After discarding the supernatant, the cell pellet was resuspended and evenly spread onto the surface of solid YEB medium supplemented with appropriate antibiotics. The plates were incubated at 28 °C for 36–48 h to form a dense bacterial lawn. Subsequently, the bacterial cells were scraped off and resuspended in an inoculation buffer (10 mM MES, 10 mM MgSO_4_, and 100 μM acetosyringone, pH 5.5–5.6). The bacterial cell density was adjusted to an OD_600_ of 0.5, and the suspension was statically incubated in the dark at 28 °C for 1 h for pre-induction prior to the inoculation treatments.

(1) Dip–inoculation method (DIP): Seedlings derived from germinated seeds were utilized as transformation recipients. The hypocotyl was excised approximately 1 cm below the cotyledonary node. The cut surface was directly dipped into the aforementioned pre-induced bacterial suspension. The seedlings were then replanted into vermiculite with the wounded surface facing upwards and co-cultivated in the dark at 28 °C for 3 days. Following co-cultivation, the inoculated *Macadamia* seedlings were transferred to a growth chamber maintained at 26 °C under a 16-h light/8-h dark photoperiod. The plants were watered daily to maintain substrate moisture, and hairy root development was evaluated at 25 days post-inoculation.

(2) Injection–inoculation method (INJ): Similarly, seedlings were employed as recipients by excising the hypocotyl 1 cm below the cotyledonary node. A 1 mL syringe was used to draw the pre-induced bacterial suspension and inject it into the cut surface of the remaining hypocotyl from multiple angles. Following the injection, the subsequent co-cultivation and maintenance procedures were identical to those described for the DIP method.

(3) Air layering-assisted in planta transformation method for mature trees: Intact living branches from 5-year-old grafted mature *Macadamia* trees were utilized as transformation recipients. Healthy, vigorous branches free of diseases, pests, and mechanical injuries were selected. The girdling treatment was performed by removing an approximately 1 cm-wide strip of phloem tissue down to the vascular cambium surface. Sterile absorbent cotton was then tightly wrapped around the girdled wound sites to facilitate liquid treatments. The experiment comprised three treatment groups: (i) *Agrobacterium* transformation group (Experimental Group)—the cotton was fully saturated with the aforementioned pre-induced bacterial suspension. (ii) Blank control group (Mock)—the cotton was saturated with an equal volume of sterile inoculation buffer. (iii) Hormone control group (Positive Control)—the cotton was saturated with an optimal concentration of commercial rooting powder solution (#100179410436, STANLEY, Shanghai, China). After the wound treatments, specialized air-layering rooting boxes were used to enclose the girdled sites and were filled with pre-sterilized cultivation substrate. Once the boxes were secured, sterile water was periodically supplemented to maintain a high-humidity microenvironment within the substrate. At 30 days post-transformation, the rooting boxes were dismantled, and the induction and development of hairy roots or adventitious roots at the wound sites were evaluated and recorded across all groups.

### 4.4. Phenotypic Characterization of Transgenic Hairy Roots

Upon the induction of hairy roots from the recipient tissues, transformation events were screened utilizing either fluorescence or white-light imaging techniques, contingent upon the specific reporter system introduced. Non-transgenic roots consistently served as negative controls across all assays.

Detection of the fluorescent reporter systems (*eGFP* and *DsRed2*): Initial fluorescence screening of the induced hairy roots was performed utilizing a handheld dual-wavelength fluorescence excitation light source (LUYOR-3415RG, LUYOR Instrument Co., Ltd., Joliet, IL, USA). Under specific excitation illumination coupled with corresponding emission filters, *eGFP*-positive hairy roots exhibited distinct green fluorescent signals, whereas *DsRed2*-positive hairy roots displayed red fluorescent signals [11,12]. The spectral characteristics of the eGFP and DsRed2 proteins correspond to excitation/emission peaks at approximately 488/507 nm and 558/583 nm, respectively. For samples presenting ambiguous signals during the preliminary screening, a fluorescence stereomicroscope (P-DSL32, Nikon, Tokyo, Japan) was deployed for high-resolution microscopic observation and image acquisition under the appropriate fluorescence channels, thereby rigorously confirming the specificity of the fluorescent signals.

Detection of the macroscopically visible reporter systems: For transformation groups harboring the *RUBY* reporter gene, specialized excitation light sources were unnecessary, as this system visualizes transformation events via the catalyzed massive accumulation of betalain pigments. Morphological observations were conducted directly under ambient white light or with a bright-field stereomicroscope; positive transgenic hairy root tissues exhibited a macroscopically visible red or dark purplish-red coloration [13,45]. Similarly, for groups transformed with the *AtPAP2* reporter gene, which functions by inducing substantial anthocyanin accumulation, transgenic hairy root tissues could be directly identified macroscopically, displaying a distinct purple or purplish-red phenotype [14]. By contrast, the non-transgenic control roots of *Macadamia* maintained their characteristic milky-white or light-brown appearance.

### 4.5. Real-Time Quantitative PCR (RT-qPCR)

To analyze the transcriptional levels of the expression cassettes within the hairy roots and calli, real-time quantitative PCR (RT-qPCR) assays were performed utilizing the SYBR Green I fluorescence intercalating dye method. Total RNA was extracted from the samples using the E.Z.N.A. Plant RNA Kit (R6827, OMEGA, Weehawken, NJ, USA). Subsequently, the integrity of the extracted RNA was evaluated via agarose gel electrophoresis, and its concentration and purity were quantified using a NanoDrop spectrophotometer, ensuring A260/280 ratios of ~2.0. Upon verification of adequate RNA quality, first-strand cDNA synthesis was executed using the HiScript III All-in-one RT SuperMix for qPCR kit (R323, Vazyme, Nanjing, China), strictly adhering to the manufacturer’s protocol for downstream quantitative analysis. The *Macadamia Actin1* gene (NCBI GenBank accession no. NC_056569) was used as the internal reference gene for RT-qPCR normalization, as it has been previously validated as a stable reference gene in macadamia [47,48]. All samples were collected from the same tissue type under identical conditions to minimize expression variation. The PCR reaction mixtures and thermocycling conditions were established according to the manual of the ChamQ Universal SYBR qPCR Master Mix kit (Q711, Vazyme, China). Relative gene expression levels were calculated using the method, with the expression level of the internal reference gene *Actin1* normalized to 1 to calibrate the expression profiles of the target genes. The specific primers utilized in this study are listed in Supplementary Table S4. The gene expression levels were calculated via the 2^−ΔΔCt^ method. All reactions were performed in triplicate.

### 4.6. Genomic DNA Extraction and PCR Confirmation

Genomic DNA was extracted from *RUBY*-positive and wild-type (non-transformed) hairy roots using the CTAB method [49]. To confirm transgene integration, genomic PCR was performed on a subset of randomly selected positive hairy roots (n = 5) using RUBY-specific primers (Table S4). The PCR program included an initial denaturation at 95 °C for 5 min, followed by 35 cycles of 95 °C for 30 s, annealing at 60 °C for 30 s, and 72 °C for 30 s, with a final extension at 72 °C for 5 min. PCR products were separated on a 1.5% agarose gel and visualized under UV light. Wild-type roots and water were used as negative controls, and the p35S-RUBY vector plasmid served as the positive control.

### 4.7. Statistical Analysis

Data acquisition across all experimental units was conducted upon the completion of the co-cultivation and induction phases. Infection-induced mortality rate = (number of dead plants post-inoculation/total number of inoculated plants) × 100%. Frequencies of positive transgenic plants (%) = (number of positive plants/total number of infected plants) × 100%. All data presented in this study are derived from a minimum of three independent biological replicates and are expressed as the mean ± standard deviation (SD). For the proportion data (e.g., infection-induced mortality rate and frequency of positive transgenic plants), the arcsine-square-root transformation was applied prior to the statistical analysis to improve normality and homoscedasticity. To assess the statistical significance among different treatment groups, one-way ANOVA followed by Tukey’s HSD post hoc test was performed for comparisons involving a single categorical variable. For experiments involving two categorical variables (inoculation method and genotype), two-way ANOVA was performed, followed by Sidak-corrected multiple comparisons for simple main effects. All statistical analyses were performed using GraphPad Prism 9 software (Version 9.5.0). Differences were defined as statistically significant at a threshold of *p* < 0.05. Untransformed percentages are presented in the figures for clarity, while statistical analyses were performed on transformed data.

## 5. Conclusions

This study successfully established an efficient, genotype-independent, and visualized in planta hairy root transformation system specifically tailored for the recalcitrant woody plant *Macadamia*. By integrating *Agrobacterium rhizogenes* with multiplexed visual reporter systems, this protocol enables rapid, antibiotic-independent screening and non-destructive identification of positive transgenic tissues under non-sterile conditions. Our findings robustly demonstrate that this in planta strategy effectively circumvents the severe genotype dependency inherent in traditional in vitro tissue culture methods, resulting in exceptional broad-spectrum transformation applicability across various open-pollinated seedlings. More crucially, coupled with the air layering technique, this study achieved in planta transformation on the branches of mature *Macadamia* trees for the first time, thereby overcoming the tissue maturity barrier that has long constrained the advancement of woody plant biotechnology.

Although this system currently primarily generates composite transgenic plants, its potential as a highly efficient in vivo testing platform is substantial. This system provides a rapid and reliable pipeline for functional validation of key candidate genes identified from large-scale transcriptomic datasets. In the future, this platform can be utilized not only to precisely assess the targeted editing efficiency of CRISPR-Cas9 and other genome-editing tools in target species, but also—when integrated with multi-omics approaches such as spatial transcriptomics and metabolomics—to elucidate the complex regulatory mechanisms underlying *Macadamia* proteoid (cluster) root development and rhizosphere microbial interactions. Furthermore, the use of rigorously validated positive hairy roots as explants, combined with continued optimization of de novo root-to-shoot organogenesis and plant regeneration pathways [35], holds promise for establishing a novel and effective strategy for the stable genetic transformation and improvement of recalcitrant woody plants.

## Figures and Tables

**Table 1:** Characteristics of the *Macadamia* spp. germplasms used in this study.

Cultivar	Parentage	Breeding Origin	Reason for Selection
A4	‘Renown’ (OP) [40]	Australia [40]	High kernel recovery and thin shell
GR1	‘8#’ (OP) [41]	Guangxi, China [41]	Local widely cultivated variety
HAES900	Unknown (OP)	Hawaii, USA [42]	International elite cultivar and thick shell
JW	Unknown (OP)	Guangxi, China	Local widely cultivated variety
O.C.	Unknown (OP)	Australia [40]	Classic commercial cultivar

OP indicates open-pollinated seedling selection.

## Data Availability

The original contributions presented in this study are included in the article/Supplementary Material. Further inquiries can be directed to the corresponding author.

## Supplementary Materials

The following supporting information can be downloaded at https://www.mdpi.com/article/10.3390/plants15162418/s1. Figure S1. PCR confirmation of *RUBY* transgene integration in transgenic hairy roots. Table S1. Seedling survival and number of positive seedlings detected at stages P1, P2, and P3 across different macadamia cultivars. Table S2. Comparative effects of two distinct inoculation treatments. Table S3. Effects of varying *A. rhizogenes* suspension concentrations on adventitious root and positive transformation efficiency. Table S4. Primers used in this study.

## References

[1] Trueman S.J. (2013). The reproductive biology of macadamia. Sci. Hortic..

[2] Hardner C.M., Wall M., Cho A. (2019). Global macadamia science: Overview of the special section. HortScience.

[3] Xia C., Jiang S., Tan Q., Wang W., Zhao L., Zhang C., Bao Y., Liu Q., Xiao J., Deng K. (2022). Chromosomal-level genome of macadamia (Macadamia integrifolia). Trop. Plants.

[4] Wang P., Mo Y., Wang Y., Fei Y., Huang J., Ni J., Xu Z.-F. (2022). Macadamia germplasm and genomic database (MacadamiaGGD): A comprehensive platform for germplasm innovation and functional genomics in *Macadamia*. Front. Plant Sci..

[5] Fei Y.-C., Mo Y., Lin K., Tao L., He X., Ye F., Xu J., Li M., Xu Z.-F. (2025). Comparative transcriptome analysis reveals a possible mechanism of starvation stress-induced fruit abscission in macadamia. BMC Plant Biol..

[6] Poudel P.D., Faveri J.-D., Topp B., Alam M. (2025). Genome-wide association studies revealed partial genetic links between early vigour and precocity in macadamia. Hortic. Res..

[7] Maharjan B.K., Islam M.T., Muzaffar A., Tschaplinski T.J., Tuskan G.A., Chen J.-G., Yang X. (2025). Woody plant transformation: Current status, challenges, and future perspectives. Plants.

[8] Ying W., Wen G., Xu W., Liu H., Ding W., Zheng L., He Y., Yuan H., Yan D., Cui F. (2023). *Agrobacterium rhizogenes*: Paving the road to research and breeding for woody plants. Front. Plant Sci..

[9] Wang P., Si H., Li C., Xu Z., Guo H., Jin S., Cheng H. (2025). Plant genetic transformation: Achievements, current status and future prospects. Plant Biotechnol. J..

[10] Jefferson R. (1989). The GUS reporter gene system. Nature.

[11] Cheng Z., Liu X., Yan S., Liu B., Zhong Y., Song W., Chen J., Wang Z., Che G., Liu L. (2023). Pollen tube emergence is mediated by ovary-expressed *ALCATRAZ* in cucumber. Nat. Commun..

[12] Sun L., Alariqi M., Zhu Y., Li J., Li Z., Wang Q., Li Y., Rui H., Zhang X., Jin S. (2018). Red fluorescent protein (DsRed2), an ideal reporter for cotton genetic transformation and molecular breeding. Crop J..

[13] Chen L., Cai Y., Liu X., Yao W., Wu S., Hou W. (2024). The RUBY reporter for visual selection in soybean genome editing. Abiotech.

[14] Duan Z.-z., Huang Z., Zhou Y.-m., Li H., Tu W., Zhang M., Yao W. (2020). Plant expression vector construction and genetic transformation of anthocyanin synthesis transcription factor *PAP2*. J. Anhui Agric. Sci..

[15] Sharifova S., Prasad K.V., Cheema A., Reddy A.S. (2025). Genetically encoded betalain-based RUBY visual reporters: Noninvasive monitoring of biological processes. Trends Plant Sci..

[16] Altpeter F., Springer N.M., Bartley L.E., Blechl A.E., Brutnell T.P., Citovsky V., Conrad L.J., Gelvin S.B., Jackson D.P., Kausch A.P. (2016). Advancing crop transformation in the era of genome editing. Plant Cell.

[17] Mwang’ingo P., Teklehaimanot Z., Lulandala L., Maliondo S. (2006). Propagating *Osyris lanceolata* (African sandalwood) through air layering: Its potential and limitation in Tanzania. S. Afr. For. J..

[18] Leakey R.R.B., Van Alfen N. (2014). Plant Cloning: Macropropagation. Encyclopedia of Agriculture and Food Systems.

[19] Zhang S.Y., Luo R.F., Wu Y.X., Zhang T.T., Yusuf A., Wang N., Li M., Duan S. (2025). Establishment and application of high-pressure propagation breeding (HPPB)-mediated genetic transformation system in citrus rootstocks. Plant Biotechnol. J..

[20] Gambino G., Gribaudo I. (2012). Genetic transformation of fruit trees: Current status and remaining challenges. Transgenic Res..

[21] De Schepper V., Steppe K., Van Labeke M.-C., Lemeur R. (2010). Detailed analysis of double girdling effects on stem diameter variations and sap flow in young oak trees. Environ. Exp. Bot..

[22] Li N., Chang Y. (2022). Effective methods for adventitious root regeneration on weeping fig stems. Forests.

[23] Spena A., Schmülling T., Koncz C., Schell J.S. (1987). Independent and synergistic activity of *rol A, B* and *C* loci in stimulating abnormal growth in plants. EMBO J..

[24] Tang M., Tao Y.-B., Fu Q., Song Y., Niu L., Xu Z.-F. (2016). An ortholog of *LEAFY* in *Jatropha curcas* regulates flowering time and floral organ development. Sci. Rep..

[25] Manuela D., Xu M. (2020). Juvenile leaves or adult leaves: Determinants for vegetative phase change in flowering plants. Int. J. Mol. Sci..

[26] Corbesier L., Vincent C., Jang S., Fornara F., Fan Q., Searle I., Giakountis A., Farrona S., Gissot L., Turnbull C. (2007). FT protein movement contributes to long-distance signaling in floral induction of *Arabidopsis*. Science.

[27] Abe M., Kobayashi Y., Yamamoto S., Daimon Y., Yamaguchi A., Ikeda Y., Ichinoki H., Notaguchi M., Goto K., Araki T. (2005). FD, a bZIP protein mediating signals from the floral pathway integrator FT at the shoot apex. Science.

[28] Tang M., Bai X., Xia Y., Huang P., Xu Z.-F. (2026). SBP-Box transcription factor *JcSPL9* regulates both seed yield and oil content in the biofuel plant *Jatropha curcas*. Plant Biotechnol. J..

[29] Tang M., Bai X., Wang J., Chen T., Meng X., Deng H., Li C., Xu Z.-F. (2022). Efficiency of graft-transmitted *JcFT* for floral induction in woody perennial species of the *Jatropha* genus depends on transport distance. Tree Physiol..

[30] Li J.J., Zhao Y.M., Liu J.F., Li C.H., Zhang H., Song Y.T., Zuo Q., Wang P.Y., Gao K., Meng D. (2026). Rooting conifer genetic research: An accessible and efficient transformation system. Plant Biotechnol. J..

[31] Brunner A.M., Nilsson O. (2004). Revisiting tree maturation and floral initiation in the poplar functional genomics era. New Phytol..

[32] Wilkie J.D., Sedgley M., Olesen T. (2008). Regulation of floral initiation in horticultural trees. J. Exp. Bot..

[33] Gitonga L., Gichuki S., Ngamau K., Muigai A., Kahangi E., Wasilwa L., Wepukhulu S., Njogu N. (2010). Effect of explant type, source and genotype on in vitro shoot regeneration in Macadamia (*Macadamia* spp.). J. Agric. Biotechnol. Sustain. Dev..

[34] Bélanger J.G., Copley T.R., Hoyos-Villegas V., Charron J.-B., O’Donoughue L. (2024). A comprehensive review of *in planta* stable transformation strategies. Plant Methods.

[35] Liu L., Qu J., Wang C., Liu M., Zhang C., Zhang X., Guo C., Wu C., Yang G., Huang J. (2024). An efficient genetic transformation system mediated by *Rhizobium rhizogenes* in fruit trees based on the transgenic hairy root to shoot conversion. Plant Biotechnol. J..

[36] Yamashita H., Daimon H., Akasaka-Kennedy Y., Masuda T. (2004). Plant regeneration from hairy roots of apple rootstock, *Malus prunifolia* Borkh. var. ringo Asami, strain Nagano No. 1, transformed by *Agrobacterium rhizogenes*. J. Jpn. Soc. Hortic. Sci..

[37] Lambers H. (2022). Phosphorus acquisition and utilization in plants. Annu. Rev. Plant Biol..

[38] Giacomello S. (2021). A new era for plant science: Spatial single-cell transcriptomics. Curr. Opin. Plant Biol..

[39] Boisson-Dernier A., Chabaud M., Garcia F., Bécard G., Rosenberg C., Barker D.G. (2001). *Agrobacterium rhizogenes*-transformed roots of *Medicago truncatula* for the study of nitrogen-fixing and endomycorrhizal symbiotic associations. Mol. Plant-Microbe Interact..

[40] Hardner C.M., Peace C., Lowe A.J., Neal J., Pisanu P., Powell M., Schmidt A., Spain C., Williams K., Janick J. (2009). Genetic Resources and Domestication of Macadamia. Horticultural Reviews.

[41] Mo Q., Qin Z., Zhao D., He X. (2007). A new macadamia cultivar—‘Guire 1’. Guangxi Trop. Agric..

[42] Hardner C. (2016). Macadamia domestication in Hawai ‘i. Genet. Resour. Crop Evol..

[43] Li C., Luo L., Fu Q., Niu L., Xu Z.-F. (2014). Isolation and functional characterization of *JcFT,* a *FLOWERING LOCUS T* (*FT*) homologous gene from the biofuel plant *Jatropha curcas*. BMC Plant Biol..

[44] Pan W., Cheng Z., Han Z., Yang H., Zhang W., Zhang H. (2022). Efficient genetic transformation and CRISPR/Cas9-mediated genome editing of watermelon assisted by genes encoding developmental regulators. J. Zhejiang Univ.-Sci. B.

[45] Lin K., Lu L.-X., Pan B.-Z., Chai X., Fu Q.-T., Geng X.-C., Mo Y., Fei Y.-C., Xu J.-J., Li M. (2025). *Agrobacterium rhizogenes*-mediated hairy root genetic transformation using *Agrobacterium* gel inoculation and RUBY reporter enables efficient gene function analysis in sacha inchi (*Plukenetia volubilis*). Int. J. Mol. Sci..

[46] Wang Y., Yang X., Wang W., Wang Y., Chen X., Wu H., Gao Z., Xu H., Liu T., Li Y. (2025). Efficient genetic transformation and gene editing of Chinese cabbage using *Agrobacterium rhizogenes*. Plant Physiol..

[47] Nock C.J., Baten A., Mauleon R., Langdon K.S., Topp B., Hardner C., Furtado A., Henry R.J., King G.J. (2020). Chromosome-scale assembly and annotation of the Macadamia genome (*Macadamia integrifolia* HAES 741). G3 Genes Genomes Genet..

[48] Yang Q., Yang Z., Zeng H., Zou M., Song X., Wan J., Wang Z., Chen J., Luo L. (2024). Evaluation and Validation of Reliable Reference Genes for Quantitative Real-Time PCR Analysis of the Gene Expression in Macadamia integrifolia. Forests.

[49] Porebski S., Bailey L.G., Baum B.R. (1997). Modification of a CTAB DNA extraction protocol for plants containing high polysaccharide and polyphenol components. Plant Mol. Biol. Report..

