# Supplementary material for: Efficient In Planta Induction of Transgenic Hairy Roots in Macadamia Seedlings and Mature Trees Using Visual Reporters
Source: Plants (Basel). 2026 Aug 7;15(16):2418. doi: 10.3390/plants15162418 (PMC13516706; doi:10.3390/plants15162418)
Supplement: Supplementary file 1 [file plants-15-02418-s001.zip › Supplementary Figure S1 PCR confirmation of RUBY transgene integration in transgenic hairy roots.pdf]

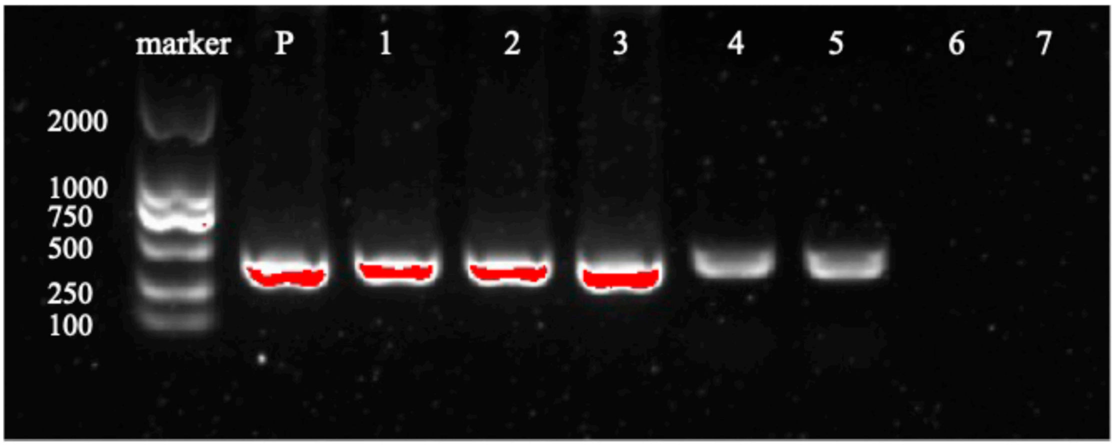

Supplementary Figure S1 PCR confirmation of *RUBY* transgene integration in transgenic hairy roots. M, DNA marker (DL2000); P, positive control (RUBY-containing plasmid); lanes 1–5, genomic DNA from representative red-pigmented (RUBY-positive) hairy roots, showing amplification of the expected 258 bp fragment; lanes 6–7, genomic DNA from wild-type (non-transformed) roots serving as negative controls, with no visible target band. The expected amplicon size is 258 bp. The results confirm stable T-DNA integration in the RUBY-positive hairy roots.
